# The association of accelerated epigenetic age with all-cause mortality in cardiac catheterization patients as mediated by vascular and cardiometabolic outcomes

**DOI:** 10.1186/s13148-022-01380-x

**Published:** 2022-12-03

**Authors:** Rong Jiang, Elizabeth R. Hauser, Lydia Coulter Kwee, Svati H. Shah, Jessica A. Regan, Janet L. Huebner, Virginia B. Kraus, William E. Kraus, Cavin K. Ward-Caviness

**Affiliations:** 1 Department of Psychiatry and Behavioral Sciences, Duke University School of Medicine, Durham, NC USA; 2 Duke Molecular Physiology Institute, Duke University School of Medicine, Durham, NC USA; 3 Department of Biostatistics and Bioinformatics, Duke University School of Medicine, Durham, NC USA; 4 Division of Cardiology, Department of Medicine, Duke University School of Medicine, Duke University, Durham, NC USA; 5 Division of Rheumatology, Department of Medicine, Duke University School of Medicine, Durham, NC USA; 6 Center for Public Health and Environmental Assessment, US Environmental Protection Agency, Chapel Hill, NC USA

**Keywords:** DNA methylation age, Accelerated epigenetic age, Aging, Cardiovascular disease, All-cause mortality, Vascular, Effect mediation

## Abstract

**Background:**

Epigenetic age is a DNA methylation-based biomarker of aging that is accurate across the lifespan and a range of cell types. The difference between epigenetic age and chronological age, termed age acceleration (AA), is a strong predictor of lifespan and healthspan. The predictive capabilities of AA for all-cause mortality have been evaluated in the general population; however, its utility is less well evaluated in those with chronic conditions. Additionally, the pathophysiologic pathways whereby AA predicts mortality are unclear. We hypothesized that AA predicts mortality in individuals with underlying cardiovascular disease; and the association between AA and mortality is mediated, in part, by vascular and cardiometabolic measures.

**Methods:**

We evaluated 562 participants in an urban, three-county area of central North Carolina from the CATHGEN cohort, all of whom received a cardiac catheterization procedure. We analyzed three AA biomarkers, Horvath epigenetic age acceleration (HAA), phenotypic age acceleration (PhenoAA), and Grim age acceleration (GrimAA), by Cox regression models, to assess whether AAs were associated with all-cause mortality. We also evaluated if these associations were mediated by vascular and cardiometabolic outcomes, including left ventricular ejection fraction (LVEF), blood cholesterol concentrations, angiopoietin-2 (ANG2) protein concentration, peripheral artery disease, coronary artery disease, diabetes, and hypertension. The total effect, direct effect, indirect effect, and percentage mediated were estimated using pathway mediation tests with a regression adjustment approach.

**Results:**

PhenoAA (HR = 1.05, *P* < 0.0001), GrimAA (HR = 1.10, *P* < 0.0001) and HAA (HR = 1.03, *P* = 0.01) were all associated with all-cause mortality. The association of mortality and PhenoAA was partially mediated by ANG2, a marker of vascular function (19.8%, *P* = 0.016), and by diabetes (8.2%, *P* = 0.043). The GrimAA-mortality association was mediated by ANG2 (12.3%, *P* = 0.014), and showed weaker evidence for mediation by LVEF (5.3%, *P* = 0.065).

**Conclusions:**

Epigenetic age acceleration remains strongly predictive of mortality even in individuals already burdened with cardiovascular disease. Mortality associations were mediated by ANG2, which regulates endothelial permeability and angiogenic functions, suggesting that specific vascular pathophysiology may link accelerated epigenetic aging with increased mortality risks.

## Background

DNA methylation (DNAm) biomarkers have emerged as widely validated estimators of biological which are both correlated with chronological age and tied to biophysiological aging [1]. Epigenetic age acceleration (AA), calculated as the difference or residual from a linear regression of DNAm age and chronological age, is associated with mortality, cancer, obesity, and several other health outcomes [2–6]. Horvath age acceleration (HAA), Phenotypic age acceleration (PhenoAA), and Grim age acceleration (GrimAA) are all well validated in their associations with mortality [7–9]. In addition, these biomarkers are associated with a host of vascular phenotypes, such as cardiovascular disease (CVD), cancer, dementia, inflammation, and hemostatic factors [1, 10, 11]. However, we still do not fully understand whether the performance of these biomarkers is impacted by pre-existing disease within individuals, nor which pathophysiologic pathways may mediate the associations. Genome-wide association studies have identified genes related to telomere lengthening [12, 13], immune function, and metabolism as potential underlying pathways driving the effects of these biomarkers. Yet, these studies have not to determine if associations among AA biomarkers and mortality are mediated by one or more specific pathways related to traditional mortality risk factors. Understanding the pathways mediating these effects will become even more important as epigenetic aging biomarkers are incorporated into clinical trials [14].

While multiple studies have associated accelerated epigenetic aging with mortality, few of these studies have focused on individuals with underlying chronic diseases, such as CVD. Such individuals have greater mortality risks than the general population, and thus mortality biomarkers may perform differently within these populations than they do in the general population. Here, we examined associations between all-cause mortality and three epigenetic age acceleration biomarkers: Horvath Age (DNAmAge), Phenotypic age (PhenoAge), and Grim age (GrimAge). DNAmAge is a multi-tissue aging biomarker based on percent methylation of 353 CpG loci present on both the Illumina 27 K and 450 K arrays [7]. PhenoAge is a blood DNAm marker constructed using 513 CpGs [8]. GrimAge is a composite mortality biomarker utilizing DNAm surrogates for plasma proteins and smoking pack years; it is estimated using the percent methylation at 1030 CpG loci as well as age and sex [9]. We chose these three epigentic biomarkers as they represent distinct approaches to quantifying the aging process through the use of DNAm. Horvath age was developed as a “pan-tissue” biomarker of the aging process and is valid in a wide range of tissue types [7]. It was one of the earliest aging biomarkers developed and is one of the most widely studied. PhenoAge was developed to track aging as reflected by several established markers of health and aging including creatinine, serum glucose, white blood cell count, and C-reactive protein [8]. As opposed to a more general biomarker of the aging process, GrimAge was developed specifically to be a mortality predictor [9]. Unlike DNAmAge, both GrimAge and PhenoAge are blood-specific aging biomarkers. We examined these associations in a cohort of individuals who had previously undergone a cardiac catheterization, to better characterize their associations with mortality in individuals already burdened with cardiovascular disease. We additionally investigated whether mechanisms related to vascular dysfunction mediated the observed associations between age acceleration biomarkers and all-cause mortality.

## Methods

### Study participants

Catheterization Genetics (CATHGEN) is a cohort of 9334 patients who underwent cardiac catheterization between 2001 and 2010 at Duke University Hospital [15], at which time, each CATHGEN participant provided informed consent for the collection of medical data and biosamples. The study was approved by the Duke University Institutional Review Board. Assessment of DNAm was performed on 562 individuals using the Illumina EPIC microarray using published methods [16]. In previous research, six neighborhood clusters (census block groups) were created in Wake, Durham, and Orange Counties in NC and were clustered based on sociodemographic characteristics [17, 18]. The individuals chosen for DNAm assessment were randomly selected from these sociodemographic clusters [19].

### DNA methylation age and age acceleration (AA)

We examined three DNA methylation age acceleration measures: HAA, PhenoAA, and GrimAA. First, genome-wide DNAm was measured using whole blood-derived DNA with Illumina Infinium MethylationEPIC BeadChips. Data processing has been previously described [20]. Background subtraction, probe dye bias correction, and stratified quantile normalization was performed using the R packages *noob* and *minfi*. CpG loci with a detection *P* value > 0.01 were removed and then any probes with > 1% missing probes across all individuals were removed. After all quality control procedures were completed, we used the resulting DNA methylation measures to estimate DNAmAge, PhenoAge, and GrimAge via the available online tool (http://dnamage.genetics.ucla.edu/). For each biomarker, AA was computed as the difference between epigenetic age and chronological age [7–9]. The residuals from regressing epigenetic age on chronological age a means of estimating age acceleration which gives age acceleration relative to the slope between chronological age and epigenetic age seen in the population. We used the difference here as an individual measure as opposed to being relative to a specific population and thus is consistent under any subgroup analyses. Additionally, we adjusted for chronological age within our models, thus removing confounding from chronological age in an identical manner as it would be to using the residuals as the indicator of accelerated aging.

### All-cause mortality

As part of the ongoing follow-up of the Duke Databank for Cardiovascular Diseases, CATHGEN participants were surveyed annually for CVD events until 2015; the participant list is compared to the National Death Index for mortality measures. The events were defined as mortality from any cause through January 31, 2019.

### Vascular and cardiometabolic phenotypes for mediation analyses

We considered a range of vascular and cardiometabolic phenotypes for the mediation analyses. The phenotypes chosen were not intended to be an exhaustive list, but chosen to represent a breadth of possible pathophysiologic pathways that could generate additional hypotheses to be tested in a more exhaustive manner in future studies. Individual-level clinical data were obtained from the history and physical examination performed by a clinician prior to the catheterization procedure and supplemented by information from the medical record. The phenotypes chosen were body mass index (BMI), systolic blood pressure (SBP), diastolic blood pressure (DBP), low density lipoprotein cholesterol (LDL-C), high density lipoprotein cholesterol (HDL-C), total cholesterol (TC), triglycerides, the number of diseased coronary vessels (NUMDZV), left ventricular ejection fraction (LVEF), coronary artery disease (CAD), peripheral arterial disease (PAD), congestive heart failure (CHF), diabetes, and plasma angiopoietin-2 (ANG2) concentrations. BMI, SBP, DBP, LDL-C, HDL-C, TC, TG were all take from medical records associated with the cardiac catheterization. NUMDZV was an ordinal variable indicating the number of occluded (diseased) coronary vessels. A vessel (artery) was defined as diseased if it had ≥ 75% occlusion for the left anterior descending, left circumflex or right coronary arteries, or ≥ 50% for the left main coronary artery considered to represent two-vessel disease. Individuals were defined as having a positive indication for CAD based on the following: The number of diseased vessels was greater than 0; a history of cardiovascular conditions or coronary artery bypass graft; a CAD index > 23 (0–100) [21, 22]; or if one of the heart vessels had a significant (≥ 75%) stenosis as determined by the interventional cardiologist. LVEF defined as the percentage of blood pumped out of the left ventricle during systole, was measured using ventriculogram at time of cardiac catheterization. If a ventriculogram was not performed, then echocardiogram, nuclear, or cardiac MRI studies within one year prior or one month after catheterization were used. History of PAD, CHF, and diabetes were assessed during the intake examination for the cardiac catheterization procedure. Stored plasma from blood collected during the cardiac catheterization procedures was used to obtain plasma concentrations of ANG2, a biomarker of vascular health. ANG2 concentrations were quantified using a sandwich immunoassay (MesoScale Discovery Cat #K151KCD-1; Gaithersburg, MD) as directed by the manufacturer. ANG2 concentrations were log-transformed to reduce skew within the distribution and approximate a normal distribution.

### Statistical analyses

**Fig. 1:**
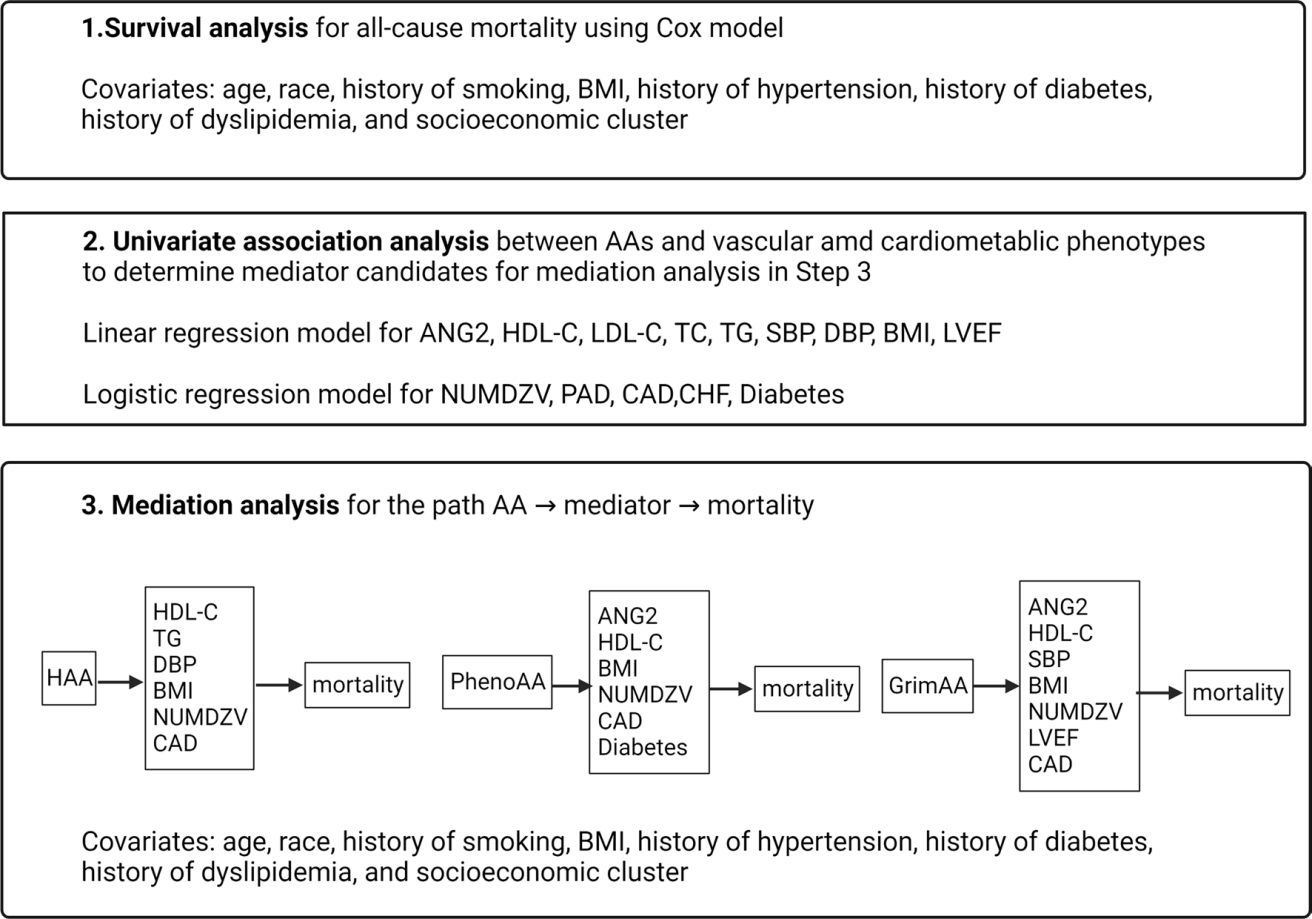
Analysis steps

Figure 1 lists the analysis steps in this study. First, we performed survival analysis for all-cause mortality associated with age acceleration as determined by the three aging biomarkers using a Cox regression model by PROC PHREG in SAS, adjusting for chronological age, sex, race, history of smoking, BMI, history of hypertension, history of diabetes, history of dyslipidemia, and socioeconomic cluster. Confounders were chosen based on previous studies using this cohort. History of smoking, hypertension, and dyslipidemia we extracted from the cardiac catheterization intake exam. Socioeconomic clusters were determined using Ward’s hierarchical clustering based on socioeconomic and demographic variables from the US Census. These have been shown to be associated with health outcomes in CATHGEN, and samples for DNA methylation were taken based on a random sampling of the clusters. Thus, including socioeconomic cluster as a confounder provided an adjustment both for socioeconomic status as well as the sampling scheme as performed in previous analyses of these data [19]. Participants who were lost to follow-up or who had not experienced an event by the last date of follow-up were coded as censored. The proportional hazards assumption was met for all models.

Next, we determined which phenotypes would be used for mediation analyses. To do this we evaluated univariate associations between the three age acceleration measures and fourteen vascular and cardiometabolic phenotypes (Table 4). These were selected to represent a range of potential pathophysiologic pathways from metabolic dysfunction (diabetes and body mass index), to vascular functioning (ANG2), and even cardiac functioning LVEF. For continuous phenotypes we used a linear regression model and for binary or ordinal variables we used logistic regression models. Only associations with at least a nominally significant *P* value (*P* < 0.05) were selected for further evaluation as potential mediators.

In the third step we evaluated the potential for mediation between the age acceleration biomarkers and mortality via the vascular and cardiometabolic phenotypes with at least nominal significance in the previous step. In the mediation analyses, we assumed the presence of both direct (age acceleration → mortality) and indirect (age acceleration → mediator → mortality) causal pathways that might provide an explanation of how age acceleration increases mortality risk. In the indirect causal pathway, the mediator variable was hypothesized to be directly associated with AA and to have a direct association with mortality. The mediation effects were estimated by the maximum likelihood method using PROC CAUSALMED with MEDIATOR in SAS. The CAUSALMED procedure uses the counterfactual framework [23], which allows for the estimation of potentially causal mediation effects by using a regression adjustment approach [24]. The CAUSALMED with MEDIATOR analysis in SAS estimated the total effect (TE, the effect of age acceleration on mortality through direct and indirect pathways), the natural direct effect (NDE, the effect of age acceleration on mortality without a mediator), and the natural indirect effect (NIE, the degree to which the effect of age acceleration on mortality was mediated by the phenotypes passing the filtering in Step 2). The TE was the sum of NDE and NIE, and the percentage of mediation was calculated as NIE/TE × 100%. Identical sets of confounders were used in the survival analysis and in the mediation analysis. The first step in the mediation analysis was to examine the interaction of each AA with the potential mediators while adjusting for the confounders. If the interaction term was not significant (*P* ≥ 0.05), we removed it and re-ran the model with main effects of the age acceleration biomarkers and mediators. Bootstrap bias-corrected 95% confidence intervals (CI) were calculated with 5000 bootstrap samples. Separate models were used for each of the three age acceleration biomarkers and nominally significant phenotypes as mediators from Step 2.

All statistical analyses were performed using the SAS software package (version 9.4, SAS Institute, Cary, NC). Given the small sample size, small number of correlated predictors, and exploratory nature of these analyses, we used a nominal *P* < 0.05 association level as an indicator of significance.

## Results

A total of 562 individuals (mean age 60.1 years, average follow-up time 7.5 years) were included in the analysis; the descriptive statistics of all variables are presented in Table 1. The study was primarily composed of women (42%) and those who self-reported as white (62%). A history of smoking was reported by 44% of study participants; there was a substantial presence of diabetes (30%) and hypertension (68%). At the end of the observation period, 40% of study participants had died. As expected, given that all study participants were referred for cardiac catheterization, the mean blood cholesterol concentration, blood pressure, and BMI were all greater than what might be observed in the general population, and LVEF was lower than expected in a general population (Table 1). The three epigenetic age biomarkers were highly correlated (Pearson r ≥ 0.80) with chronological age (Table 2) and were also correlated with each other (r ≥ 0.76).

**Table 1:** Characteristics of the study cohort

Variable	*N*	Mean	SD
Age (years)	562	60.10	12.42
Angiopoeitin-2 (pg/mL)	532	4742.68	3897.90
Left ventricular ejection fraction (%)	559	56.64	13.07
Body mass index (kg/m^2^)	562	30.80	7.64
HDL cholesterol (mg/dL)	429	49.06	14.74
LDL cholesterol (mg/dL)	397	106.55	41.67
Total cholesterol (mg/dL)	430	185.13	48.40
Triglycerides (mg/dL)	421	160.28	133.70
Systolic blood pressure (mmHg)	562	145.77	24.34
Diastolic blood pressure (mmHg)	562	82.15	13.77
DNAmAge (years)	562	64.90	10.89
PhenoAge (years)	562	51.26	12.39
GrimAge (years)	562	57.91	10.32
Horvath age acceleration (years)	562	4.79	6.60
Phenotypic age acceleration (years)	562	− 8.85	7.73
Grim age acceleration (years)	562	− 2.20	6.45

Variable	Total	*N*	Percent (%)
Sex (Women)	562	234	41.64
Race (Caucasian)	562	348	61.92
History of Smoking	562	248	44.13
All-cause mortality	562	223	39.68
Peripheral artery disease	562	33	5.87
Congestive heart failure	546	145	26.56
Coronary artery disease	557	299	53.68
History of hypertension	562	384	68.33
History of diabetes	562	170	30.25
History of dyslipidemia	562	323	57.47

**Table 2:** Pearson correlation between epigenetic age biomarkers and chronological age

	Age	DNAmAge	PhenoAge	GrimAge
Age	1	0.85	0.81	0.86
DNAmAge	0.85	1	0.86	0.76
PhenoAge	0.80	0.86	1	0.79
GrimAge	0.86	0.76	0.79	1

For all three AA biomarkers, greater age acceleration was significantly associated with an increased hazard for all-cause mortality (Table 3). In the CATHGEN population, with a high prevalence of CVD, the estimated HR of HAA was the smallest in magnitude (HR = 1.03, 95% CI= 1.01, 1.05), followed by the HR for PhenoAA (HR = 1.05, 95% CI= 1.03, 1.07), with the HR of GrimAA being the largest in magnitude (HR = 1.10, 95% CI=1.07, 1.13). Stated another way for each additional year of HAA the risk of all-cause mortality increased by 3% (95% CI = 1–5%) while for PhenoAA the increase in all-cause mortality risk per additional year was 5% (95% CI = 3–7%) and for GrimAA the increase in all-cause mortality risk per additional year was 10% (95% CI = 7–13%).

**Table 3:** Survival analysis of epigenetic age accelerations with all-cause mortality

	HR (95% CI)	*P*
Horvath age acceleration	1.03 (1.01, 1.05)	0.01
Phenotypic age acceleration	1.05 (1.03, 1.07)	< 0.0001
Grim age acceleration	1.10 (1.07, 1.13)	< 0.0001

Shown in this table are associations between the three age acceleration biomarkers and all-cause mortality using Cox proportional hazards models. Models were adjusted for chronological age, sex, race, history of smoking, BMI, history of hypertension, history of diabetes, history of dyslipidemia, and socioeconomic cluster as described in the “Methods” section

*CI* Confidence interval, *HR* Hazard ratio

Table 4 gives the *P* values for the univariate associations between the accelerated aging biomarkers and the 14 vascular and cardiometabolic phenotypes considered as potential confounders. Only measures having at least a nominally significant (*P* < 0.05) association with an AA biomarker (shown in bold in Table 4), were continued forward to be examined for evidence of mediation. The first step of the mediation analysis involved testing for significant interactions between the age acceleration biomarkers and potential mediators in association with all-cause mortality. We observed no significant interactions and thus proceeded with the mediation analysis as described in the Methods.

**Table 4:** *P* values of the association between AAs and potential mediators using univariate regression models

	HAA	PhenoAA	GrimAA
Angiopoietin-2	0.65	** < 0.0001** +	**0.0001** +
HDL cholesterol	** < 0.0001** −	**0.001** −	** < 0.0001** −
LDL cholesterol	0.95	0.97	0.29
Total cholesterol	0.34	0.76	0.97
Triglyceride	**0.04** +	0.14	0.23
Systolic blood pressure	0.15	0.06	**0.002** −
Diastolic blood pressure	**0.007** +	0.37	0.10
Body mass index	**0.0003** +	**0.001** +	**0.0004** +
Number of disease vessels	**0.0007** −	**0.02** −	**0.003** −
Left ventricular ejection fraction	0.98	0.05	**0.008** −
Peripheral artery disease	0.38	0.41	0.19
Coronary artery disease	** < 0.0001** −	**0.009** −	**0.03** −
Congestive heart failure	0.10	0.41	0.78
Diabetes	0.10	**0.002** +	0.16

Bold indicates nominally significant at *P* < 0.05; + indicates a positive univariate association; − indicates a negative univariate association

**Fig. 2:**
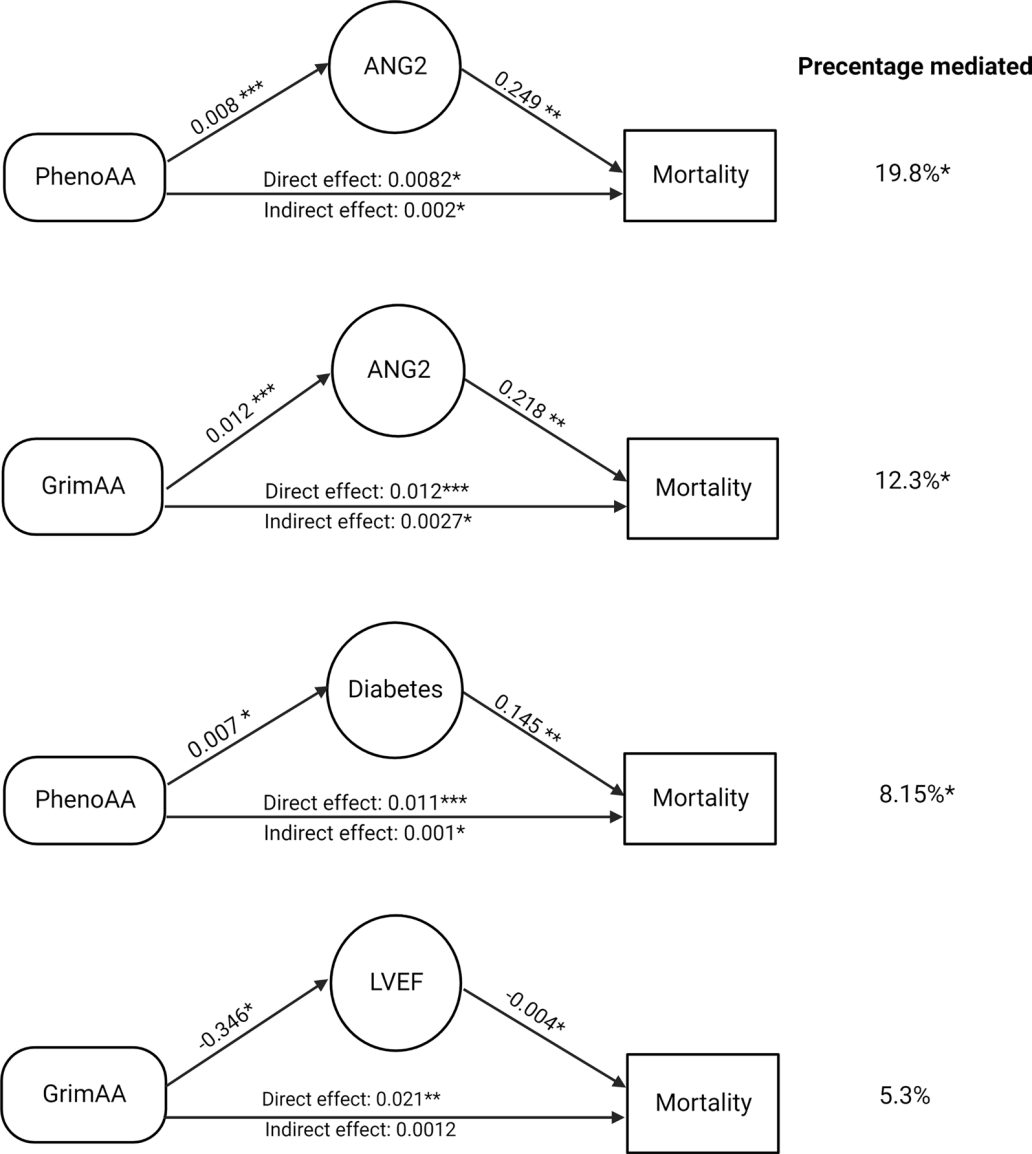
The direct and indirect pathway effect of epigenetic age acceleration to mortality via mediators (***P < 0.0001, **P < 0.001, *P < 0.05). The estimates of the mediator effect were shown on the arrows for each step

The mediation effects are presented in Table 5, summarizing percentage mediated, TE, NDE, and NIE. Both TE and NDE of for all age acceleration biomarkers showed significant, positive associations with mortality, matching what was seen in the initial Cox proportional hazards models. There were three significant (*P* < 0.05) NIEs of mediation pathways: PhenoAA to ANG2 on mortality (*P* = 0.004); PhenoAA to diabetes to mortality (*P* = 0.034); and GrimAA to ANG2 to mortality (*P* = 0.009). The indirect path of GrimAA to LVEF to mortality narrowly missed the nominally significant *P* value (NIE *P* = 0.057). Figure 2 illustrates the effect estimations of AAs to mediators for mortality through the direct and indirect pathways, and the estimated percentage mediated, while Table 5 gives the TE, NDE, and NIE for those pathways with significant mediation (NIE). The full list of all mediation pathways tested is given in Table 6.

**Table 5:** Summary of percentage mediated, total effect, direct effect, and indirect effect of AAs to mortality through mediators for those mediation analyses with significant (*P* < 0.05) mediation

Pathway	AA biomarker	PhenoAA	PhenoAA	GrimAA	GrimAA*
	Mediator	ANG2	Diabetes	ANG2	LVEF*
Percentage mediated	Estimate	19.8	8.15	12.3	5.277
	95% Lower	6.69	1.52	3.4	0.812
	95% Upper	48.483	22.822	26.933	15.613
	*P*	0.016	0.043	0.014	0.065
Total effect (TE)	Estimate	0.01	0.012	0.022	0.022
	95% Lower	0.005	0.007	0.015	0.015
	95% Upper	0.016	0.017	0.029	0.029
	*P*	< 0.0001	< 0.0001	< 0.0001	< 0.0001
Natural direct effect (NDE)	Estimate	0.008	0.011	0.019	0.021
	95% Lower	0.003	0.006	0.012	0.013
	95% Upper	0.014	0.016	0.026	0.028
	*P*	0.002	< 0.0001	< 0.0001	< 0.0001
Natural indirect effect (NIE)	Estimate	0.002	0.001	0.0027	0.0012
	95% Lower	0.0008	0	0.0008	0.0002
	95% Upper	0.0038	0.002	0.0054	0.0031
	*P*	0.004	0.034	0.009	0.057

The complete results from the mediation analysis are given in Table 6

*The mediation analysis for GrimAA to LVEF to mortality was marginally significant (indirect effect *P* = 0.057)

**Table 6:** Summary of total, direct, indirect and mediated effect results of AAs to mortality through mediators

Pathway		Percentage mediated				Total effect (TE)				Natural direct effect (NDE)				Natural indirect effect (NIE)			
AA	Mediator	Estimate	95%CI		*P*	Estimate	95%CI		*P*	Estimate	95%CI		*P*	Estimate	95%CI		*P*
Biomarker			Lower	Upper			Lower	Upper			Lower	Upper			Lower	Upper	
HAA	HDL	− 0.049	− 8.922	9.164	0.987	0.012	0.005	0.019	0.001	0.012	0.005	0.019	0.001	− 0.000006	− 0.0009	0.0009	0.987
HAA	Trigly	− 1.392	− 14.450	2.048	0.542	0.012	0.005	0.019	0.001	0.012	0.005	0.019	0.001	− 0.00017	− 0.0013	0.0002	0.530
HAA	DBP	− 0.615	− 14.124	2.134	0.666	0.009	0.003	0.015	0.006	0.009	0.003	0.015	0.005	− 0.00005	− 0.0008	0.0002	0.661
HAA	BMI	− 0.724	− 16.968	2.089	0.635	0.009	0.003	0.015	0.006	0.009	0.003	0.015	0.005	− 0.00006	− 0.0008	0.0002	0.629
HAA	NUMDZV	0.769	− 3.483	12.295	0.711	0.010	0.004	0.016	0.003	0.009	0.004	0.016	0.003	0.00007	− 0.0003	0.0009	0.711
HAA	CAD	− 2.093	− 19.996	4.708	0.577	0.009	0.003	0.015	0.004	0.009	0.003	0.016	0.003	− 0.0002	− 0.001	0.0004	0.562
PhenoAA	ANG2	19.781	6.688	48.483	**0.016**	0.010	0.005	0.016	< .0001	0.008	0.003	0.014	0.002	0.002	0.0008	0.0038	**0.004**
PhenoAA	HDL	0.028	− 4.515	5.645	0.986	0.013	0.007	0.019	< .0001	0.013	0.007	0.019	< .0001	0.000004	− 0.0005	0.0007	0.986
PhenoAA	BMI	− 0.432	− 7.229	1.098	0.638	0.011	0.006	0.016	< .0001	0.011	0.006	0.016	< .0001	− 0.00005	− 0.0007	0.0001	0.636
PhenoAA	NUMDZV	0.067	− 3.467	5.138	0.961	0.011	0.006	0.016	< .0001	0.011	0.006	0.016	< .0001	0.000008	− 0.0004	0.0005	0.961
PhenoAA	LVEF	6.089	0.924	17.785	0.093	0.011	0.006	0.016	< .0001	0.011	0.006	0.015	< .0002	0.0007	0.000	0.002	0.084
PhenoAA	CAD	− 0.620	− 7.942	3.785	0.785	0.011	0.006	0.016	< .0001	0.011	0.006	0.016	< .0001	− 0.0001	− 0.001	0.0004	0.783
PhenoAA	Diabetes	8.153	1.520	22.822	**0.043**	0.012	0.007	0.017	< .0001	0.011	0.006	0.016	< .0001	0.0010	0.000	0.002	**0.034**
GrimAA	ANG2	12.264	3.402	26.933	**0.014**	0.022	0.015	0.029	< .0001	0.011	0.012	0.026	< .0001	0.0027	0.0008	0.0054	**0.009**
GrimAA	HDL	− 0.659	− 7.671	4.096	0.762	0.023	0.015	0.031	< .0001	0.023	0.015	0.031	< .0001	− 0.00015	− 0.0016	0.0009	0.762
GrimAA	SBP	0.074	− 1.795	3.218	0.920	0.021	0.014	0.029	< .0001	0.021	0.014	0.029	< .0001	0.000016	− 0.0004	0.0006	0.920
GrimAA	BMI	− 0.115	− 3.388	1.175	0.812	0.021	0.014	0.028	< .0001	0.021	0.014	0.028	< .0001	− 0.00002	− 0.0007	0.0002	0.812
GrimAA	NUMDZV	1.166	− 0.535	7.502	0.379	0.022	0.015	0.029	< .0001	0.022	0.014	0.029	< .0001	0.00026	− 0.0001	0.0014	0.376
GrimAA	LVEF	5.277	0.812	15.613	0.065	0.022	0.015	0.029	< .0001	0.021	0.013	0.028	< .0001	0.0012	0.0002	0.0031	0.057
GrimAA	CAD	2.952	− 0.045	11.140	0.164	0.022	0.015	0.029	< .0001	0.021	0.014	0.029	< .0001	0.001	− 0.00001	0.002	0.157

Bold indicates nominally significant at *P* < 0.05

The greatest degree of mediation, as indicated by the percent mediation was seen for PhenoAA and ANG2 where ANG2 mediated 19.8% (95% CI = 6.7, 48.5, *P* = 0.02) of the association between PhenoAA and mortality. ANG2 also mediated associations between GrimAge and mortality though the percent mediation (12.3%; 95% CI = 3.4, 26.9; *P* = 0.01) was lower than that seen for PhenoAA. The final significant mediation was seen for GrimAA and diabetes where the presence of diabetes was estimated to mediate 8.15% (95% CI = 1.52, 22.8; *P* = 0.04) of the association between GrimAA and mortality. While many other associations were seen between mediators and mortality as well as between age acceleration biomarkers and mediators after adjusting for confounders (Table 6) only these three pathways showed evidence of mediation between aging biomarkers and mortality.

## Discussion

We observed that all three AAs were significantly associated with mortality in a cardiac catheterization population. Though these biomarkers are widely accepted to be associated with mortality [2], they have seldom been evaluated in a population enriched for CVD who therefore have elevated mortality rates as compared to the general population. In CATHGEN, PhenoAA and GrimAA were more strongly associated with all-cause mortality than HAA, which is consistent with observations from population-based studies of healthy individuals [9].

The association between mortality and PhenoAA was mediated by ANG2 and diabetes; this implies that vascular and metabolic dysfunction may be central to the mechanisms connecting mortality and accelerated phenotypic age. Associations between GrimAA and mortality were also mediated by ANG2, and marginally by LVEF, suggesting a strong role for vascular dysfunction in GrimAA-mortality associations. ANG2 is a potent biomarker of vascular dysfunction and atherosclerosis in general and is highly predictive of PAD specifically, which may serve as a more proximal endophenotype of vascular remodeling. ANG2 is a member of the angiopoetin-Tie2 signaling pathway that competes directly with angiopoietin-1 at the Tie2 receptor. Increased ANG2 concentrations may represent increased responsiveness to inflammatory stimuli [25]. ANG2 concentrations have been shown to be increased in individuals with CVD [26, 27] and in individuals with PAD [28]. However, we didn’t observe an association with PAD, possibly driven by the small number of PAD cases (N=33). Associations between PhenoAA and mortality were also mediated by diabetes (8.15% mediation), while there was some evidence for mediation of the GrimAA mortality associations by LVEF (5.3% mediation) this did not achieve statistical significance. No significant mediation was found for HAA.

Observing significant mediation amongst PhenoAA and GrimAA, but not HAA, in part might be explained by their construction. HAA was constructed by modeling the relationship between DNA methylation and chronological age in a wide variety of tissues and cell types. In doing so, it captures many age-related effects, and is associated with a variety of age-related outcomes like increased clotting factors [29], inflammation [30], renal dysfunction [31], frailty [32], heart disease [33], and lung disease [34]. However, as it incorporated a range of cell types the mechanisms that mediate its associations with mortality may be even more basic and conserved across cell types than the ones examined here. In contrast, both PhenoAge and GrimAge are blood-specific biomarkers designed to capture some intermediate phenotypes related to aging and mortality risk. GrimAge was constructed in part using intermediate epigenetic biomarkers for adrenomedullin, beta-2 microglobulin, cystatin C, growth differentiation factor 15, leptin, plasminogen activation inhibitor 1, and tissue inhibitor metalloproteinase 1 [9]. PhenoAge was constructed in parting using methylation biomarkers capturing aging-relevant changes in albumin, creatinine, serum glucose, C-reactive protein, blood lymphocyte percentage, mean cell volume, red blood cell distribution width, alkaline phosphatase concentrations, and white blood cell counts [8]. The inclusion of serum glucose-associated epigenetic signals in PhenoAA may explain the partial mediation of its mortality associations by diabetes. However, this study is the first to quantify such mediation, while also validating PhenoAA-mortality associations in a population enriched for individuals with CVD. Interestingly, associations between PhenoAA and mortality were even more strongly mediated by ANG2 than diabetes, though biomarkers of vascular dysfunction were not utilized in the estimation of PhenoAge whereas the primary diabetes indicator (serum glucose) was.

This study has several novel components. First, it is among the first few studies to evaluate associations among multiple epigenetic aging biomarkers and mortality in a population enriched for CVD. Typically, these biomarkers are evaluated in the general population, leaving open the question regarding their validity for predictions in populations with elevated mortality risks, like those with substantial underlying CVD. Second, this study estimated the percent of the mortality associations mediated by several common pathways, including ones related to vascular health (ANG2, SBP, DBP), cardiovascular (LVEF, CAD), and metabolic disease (blood cholesterol, diabetes). The observation that vascular health may be a significant mediating pathway, even beyond diabetes, which was more explicitly captured in the construction of the biomarkers, is of potential clinical relevance. Though this study’s sample size was relatively small, these results provide the first estimates of the proportion of the epigenetic aging-mortality associations that are mediated by several relevant clinical pathways. Additionally, as these aging biomarkers become incorporated into clinical trials to evaluate evidence of aging related effects of interventions [14], it will be important to understand the pathophysiological pathways most likely to be altered in conjunction with any related health effects. An additional strength of this paper was our use of a causal mediation analysis approach, which may help to generate robust, generalizable estimates.

The sample size of the present analyses limited the ability for more precise estimates and the examination of race- and sex-specific effects. Given our relatively small sample size and the secondary nature of these analyses, we considered our analyses of mediation to be a first, exploratory examination; this is reflected in our choice to use a nominal *P* value cutoff of *P* < 0.05 to determine significance. Additionally, given the correlation amongst the statistical hypotheses being tested, we considered a nominal *P* value of 0.05 for significant effects to be reasonable. We believe these results still provide useful, novel information that can be built upon in future studies with larger sample sizes and greater resolution of the vascular and metabolic phenotyping. Another limitation of the study is that only a single assessment timepoint was available. With longitudinal data it might be possible to evaluate both the degree to which accelerated epigenetic aging is a risk factor for chronic diseases such as diabetes and the degree to which chronic disease may accelerate the aging process. Adding this additional causal path to evaluations of accelerated aging, chronic disease, and mortality risk will allow for further understanding of these biomarkers and their relationship to chronic disease. This will require more longitudinal studies with assessments of both DNA methylation and chronic disease at multiple timepoints. Since many patients referred for catheterizations studies at Duke are cared for outside our outpatient treatment geographic region, we did not have access to the complete medical history for many of the participants; this limited our ability to adjust for other potentially relevant confounders, such as medication use, lifestyle, and behavioral factors. The socioeconomic status variable used in this analysis was based on a cluster analysis of census block group data. This approach potentially allows for the cumulative impacts of socioeconomic status to be explored; however, it lacks the resolution of individual-level collected socioeconomic status data, which were unavailable in this dataset.

## Conclusions

This study provides evidence that epigenetic age acceleration strongly predicts mortality in a sample of individuals enriched for underlying CVD. The associations in these populations may be mediated by pathophysiologic pathways related to vascular function and metabolic health. Although accelerated aging biomarkers are not yet used in clinical practice they are being increasingly incorporated into clinical trials [14]. This study provides clinical researchers with further context on the pathophysiologic pathways that underlie associations between these biomarkers and mortality, while also validating mortality association in a population enriched for CVD. Further studies are needed with larger sample sizes to replicate the mediation pathways between accelerated epigenetic aging and mortality.

## Data Availability

The data used and/or analyzed during the current study are available from the corresponding author on request.

## Abbreviations

AA: Age acceleration
ANG2: Angiopoeitin-2
BMI: Body mass index
CAD: Coronary artery disease
CHF: Congestive heart failure
CVD: Cardiovascular disease
DBP: Diastolic blood pressure
DNAm: DNA methylation
DNAmAge: Horvath age
GrimAA: Grim age acceleration
GrimAge: Grim age
HAA: Horvath age acceleration
HDL-C: HDL cholesterol
LDL-C: LDL cholesterol
LVEF: Left ventricular ejection fraction
NDE: Natural direct effect
NIE: Natural indirect effect
NUMDZV: Number of diseased coronary vessels
PAD: Peripheral artery disease
PhenoAA: Phenotypic age acceleration
PhenoAge: Phenotypic age
SBP: Systolic blood pressure
TC: Total cholesterol
TE: Total effect
TG: Triglyceride
